# The proximity of a highway increases CO_2_ respiration in forest soil and decreases the stability of soil organic matter

**DOI:** 10.1038/s41598-021-00971-7

**Published:** 2021-11-03

**Authors:** Dawid Kupka, Mateusz Kania, Piotr Gruba

**Affiliations:** Department of Forest Ecology and Silviculture, University of Agriculture in Kraków, Al. 29 Listopada 46, 31-425 Kraków, Poland

**Keywords:** Carbon cycle, Geochemistry, Forest ecology

## Abstract

Roadways traverse many forest areas and they often have harmful effects on forest soils, including the modified stability of soil organic matter (SOM). Soil CO_2_ respiration is an important indicator of SOM biological stability. The aim of this study was to test the hypotheses that a roadway will (1) modify the composition of the cation exchange capacity of adjacent forest soils, and (2) significantly decrease the stability of SOM. Two study sites were established in Scots pine and Silver fir stands, located close to the S7 highway in central Poland, which was opened to traffic in 1984. From each site, samples were taken at 2, 12 and 22 m from the forest edge. Soil CO_2_ respiration was determined using closed chamber incubation with an alkali trap. We also conducted a comprehensive analysis of soil chemical properties. The stoichiometric ratios of chosen chemical parameters to total carbon (C_t_) were calculated. In both sites, we observed increased soil pH and CO_2_ respiration in the vicinity of the highway, as well as increased ratios of exchangeable calcium (Ca), magnesium (Mg) and sodium (Na) to C_t_. In the fir site, the humic and fulvic acids, the dissolved organic carbon (DOC) content and aluminum (Al) to C_t_ ratio were depleted in close proximity to the highway. We suggest that the combined effect of Ca and Na ions, originating from winter de-icing, caused the depletion of Al and hydrogen (H) in the soil close to the forest edge and, therefore, resulted in lower SOM stability expressed as the decreased DOC and pyrophosphate-extractable carbon content, as well as the release of CO_2_. We conclude that the changes of SOM stability with distance were the effect of modification of ion-exchange relationships (particularly base cations versus Al^3+^ with H^+^) rather than forest stand species or intrinsic SOM properties (like functional groups, the recalcitrance of bindings etc.). Our work supports earlier studies, confirming the significant impact of Al and H on SOM stability.

## Introduction

Growing awareness of the potential to mitigate global climate change through the sequestration of soil organic carbon (SOC) makes it important to thoroughly investigate the mechanisms of SOC destabilization. The global pool of SOC is estimated to be about 1500 Pg C (out of a total soil carbon pool of 2500 Pg). The soil C pool is much larger than either the biotic (560 Pg) or atmospheric (760 Pg) C pools^1^. The highest carbon-rich terrestrial ecosystems are forests, whose soils contain two-thirds of the global C stock^2^. SOC is the quantifiable fraction of soil organic matter (SOM). The stability of SOM can be defined as the resistance to any disturbing factors. There are biological, physical (including thermal) and chemical SOM stability. The biological SOM stability can be expressed either as the soil microbial respiration rate or intensity or as the SOC content (either total C or its particular fractions)^3^. Soil respiration is considered to be a key process in releasing C from soils as carbon dioxide (CO_2_)^4^. The production of CO_2_ in forest soils is the result of two processes: SOM decomposition and root respiration^5^. Soil respiration is recognized as one of the largest fluxes in the global C cycle, and its magnitude is estimated as a range of 60–100 Pg C year^−1^. Only net primary production has a larger flux, in the magnitude of 100–120 Pg C year^−1^^6,7^. Given the importance of the link between the soil CO_2_ respiration and the SOM stability, a key challenge is to get a precise understanding of all the factors that influence SOM stability and the mechanisms of these influences.

There are numerous factors responsible for SOM stability. The relationship of several factors to C stabilization in forest soils has been thoroughly documented, such as temperature, forest type, soil type, soil microbial population, geographic location, and elevation^8^. The principal soil parameter, which is soil texture (expressed as the particle size distribution), also has a considerable impact on SOM^9–11^. However, there are still factors whose influence on SOM stability in forest soils has not been fully documented or remains a matter of debate owing to uncertainty regarding their impact. These include, among others, acidity [mainly aluminum (Al) chemistry], and tree species. The main discussion on the impact of these factors is whether they contribute to SOM stabilization directly or indirectly. Mueller et al.^12^ found that the chemistry of C, N and hydrolyzing cations in mineral forest soils depended on the tree species because they resulted from the different chemical composition of roots and leaves. However, parent material, topography and climate characteristics are the main factors influencing soil biogeochemistry. Al in soils can influence SOM stability. Increased levels of Al in forest soils contributed to a decrease in the SOM decomposition rate of about 35%^13^. Another important effect of Al is a reduction in the leaching of dissolved organic C (DOC). Soil pH is also significantly correlated with C storage in forest soils. Based on a considerable database from forest inventory sites in Poland, the largest organic C stocks were found in all horizons in the pH range ~ 3.4–4.0^14^.

When considering the relationship between the stability of SOM and the factors affecting it, one should also consider the constantly growing anthropogenic pressure. Human activities can lead to the emergence of emission sources from substances that modify the chemistry of forest soils. One of these is the expanding network of highways, which are an important zonal source of pollution^15^. As Ampoorter et al.^16^ have shown, the amount of CO_2_ released from forest soils can be a valuable indicator of physical soil degradation. However, it is still debatable whether the intensity of soil respiration is an indicator of the chemical degradation of soil. Highways traverse a considerable proportion of forest areas and exert many harmful effects. One of the most important environmental impacts of highways in cold climates is the winter maintenance process, during which de-icing is applied using various agents. The most commonly used de-icing agent is sodium chloride (NaCl), which has been used since the 1940s^17–19^. In the winter road maintenance process in Poland, NaCl or mixtures of NaCl with calcium chloride (CaCl_2_) are used. The most common ratios of NaCl to CaCl_2_ are 4:1, 3:1 or 2:1. CaCl_2_ is an effective de-icing agent at temperatures below − 10 °C. It rapidly absorbs moisture, accelerating the de-icing effect of NaCl^20^. The annual usage of de-icing salts is considerable. For example, 17 million tons are used annually in the USA, 6 million tons in Canada, and 2 million tons in France^21^. In Poland, approximately 300 thousand tons of NaCl and 2.3 thousand tons of CaCl_2_ are used during a single winter season^22,23^.

The aim of this study was to investigate the influence of highway proximity on the stability of SOM in adjacent soils of temperate forests. We assumed that the soil chemistry, particularly the composition of the cation exchange capacity (CEC), in the vicinity of a road would be considerably modified. We hypothesized that this change would affect the accumulation and stability of SOM. In this work we examined the SOM characteristics of the mineral topsoil of two adjacent forests, being traversed by the S7 highway in central Poland. Research has been carried out including a column experiment, based on the measurement of soil CO_2_ respiration as well as comprehensive physiochemical analyses. We tested the following hypotheses: (1) the proximity of the highway will modify the composition of CEC of adjacent forest soils, and (2) this would significantly decrease the stability of SOM.

## Materials and methods

### Study site and soil sampling

**Figure 1:**
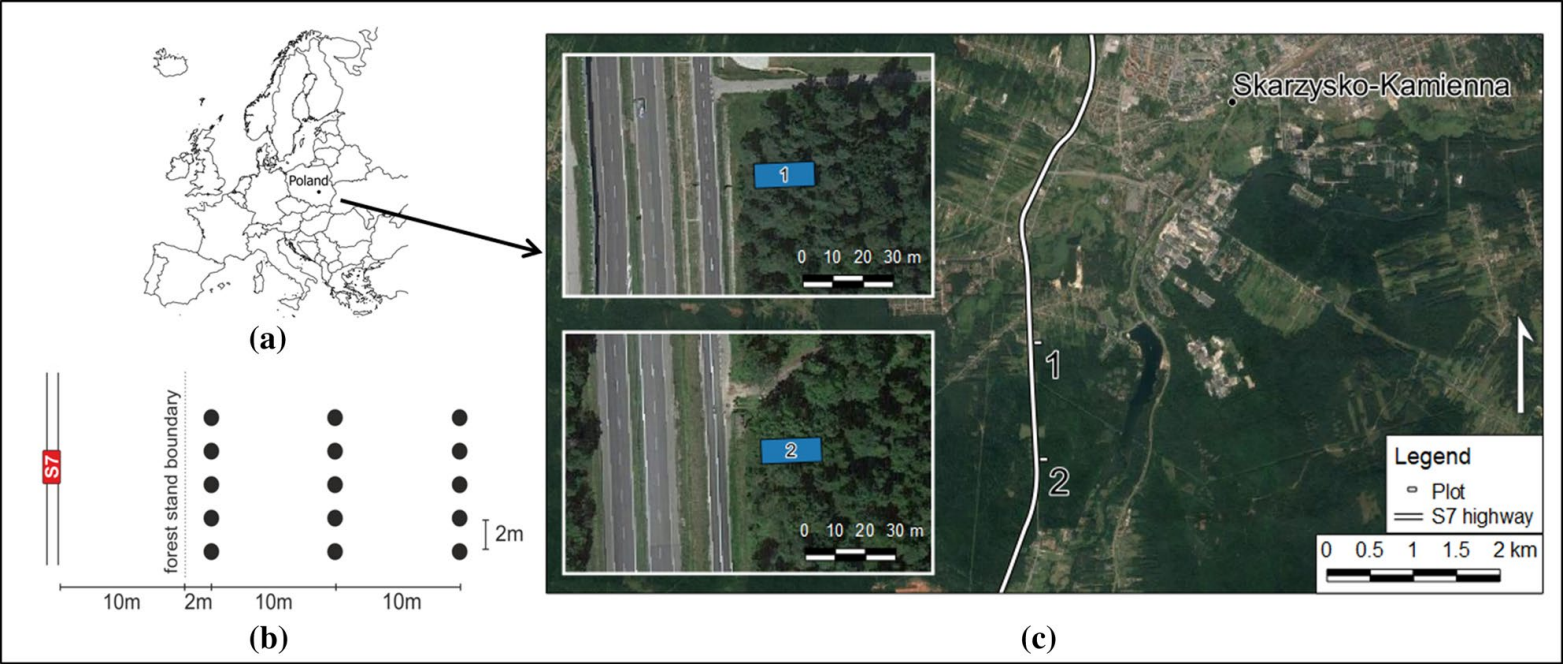
Location of the study site: (**a**) location of Poland in Europe; (**b**) sampling scheme; (**c**) location of study sites. Maps (**a**) and (**c**) were generated using QGIS software^25^ (QGIS, Version 2.18.25; http://www.qgis.org).

Two study sites were established in forest stands located in the vicinity of the S7 highway, between the cities of Skarzysko-Kamienna and Kielce in central Poland (Fig. 1). The highway was opened to traffic in 1984. The annual average daily traffic volume for this section of highway was 21,422 vehicles per day in 2015^24^.

The terrain at the study site is relatively flat, the elevation was about 250 m above sea level. The climate conditions of the investigated area are characterized as intermediate between subcontinental and Central European^26^. The average annual temperature in the years 1991–2005 was 7.8 °C, whilst the annual precipitation was 662 mm on average^27^.

**Figure 2:**
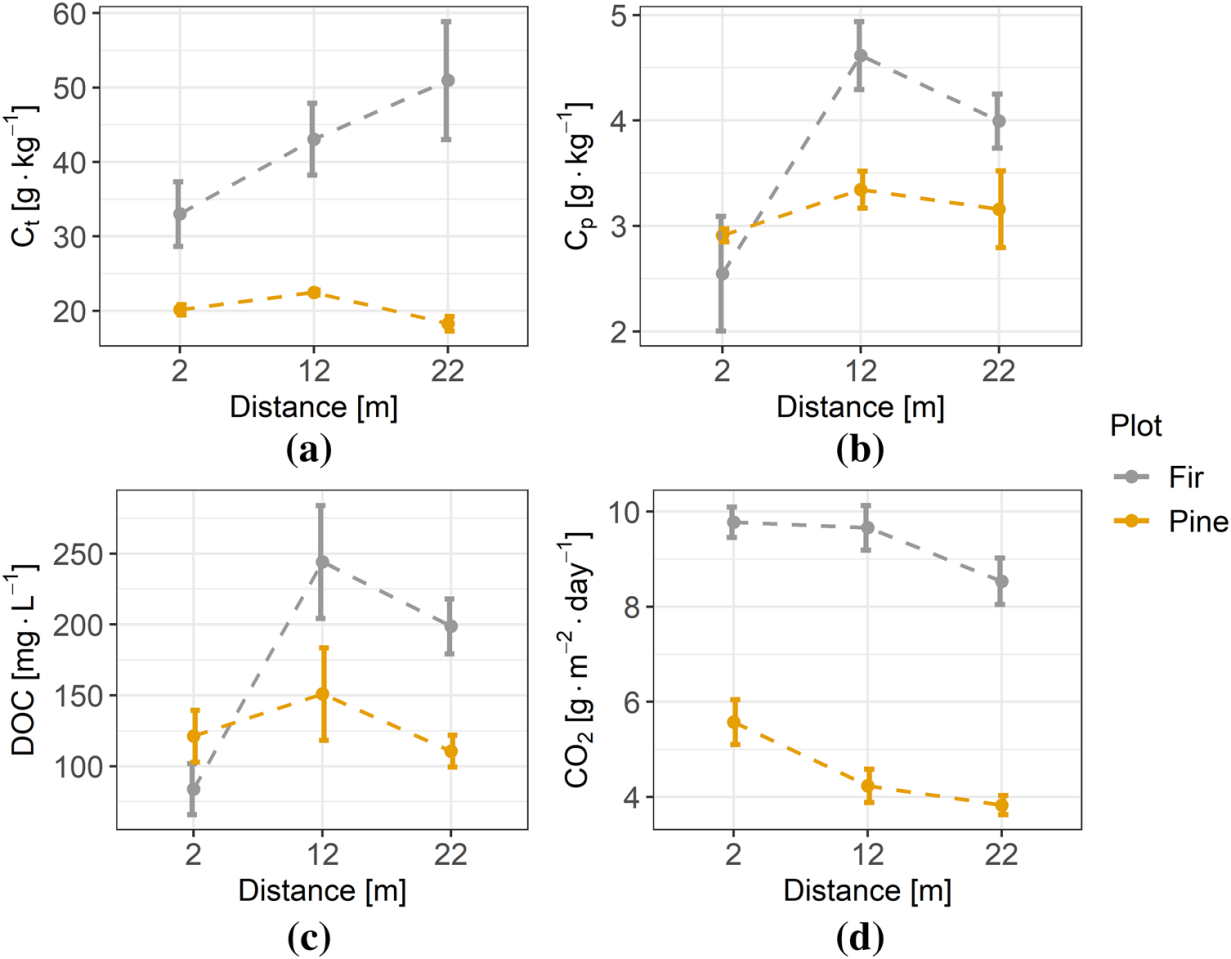
Total carbon (C_t_) (**a**), pyrophosphate extractable carbon (C_p_) (**b**), dissolved organic carbon (DOC) (**c**), and soil CO_2_ respiration (**d**) plotted against the distance from the forest edge. The circle represents the mean; the whiskers indicate the standard error.

Both sites boarded the forest stand edge, as shown in Fig. 2b,c. Between the edge of the stand in both sites and road edge is 10 m of grass verge. Sites differed in tree species, stand age, soil type and parent material. Both sites are characterized by a multistoried stand with developed understory and undergrowth vegetation. Detailed characteristics are given in Table 1.

**Table 1:** Characteristics of the study plots.

	Site 1	Site 2
Coordinates	51° 05′ 12.9″ N 20° 50′ 12.3″ E	51° 04′ 29.3″ N 20° 50′ 14.0″ E
Species	Scots pine (*Pinus sylvestris* L.)	Silver fir (*Abies alba* Mill.)
Forest stand age (years)	52	64
Soil unit	Albic Podzol	Dystric Cambisol
Parent material	Quaternary sandstone	Triassic sandstone

From each site, during the autumn of 2019, 15 samples were taken at three distances (2, 12 and 22 m) from the forest edge, according to the sampling scheme shown in Fig. 1b. Sampling was performed in October, prior to the beginning of the road winter de-icing process. The samples were taken from the top 10 cm of mineral (A) topsoil, after removing away the organic (O) horizon. The soil was collected using round plastic columns, 15 cm in height and 10.5 cm in diameter.

### Measurement of soil CO_2_ respiration

The amount of carbon dioxide (CO_2_) released from the soil was assessed by performing closed chamber incubation with sodium hydroxide (NaOH) trap^28^. A beaker with 30 mL of 1 M NaOH was placed on the top of each column of soil. CO_2_ released from soil was converted into Na_2_CO_3_ according to Eq. (1):

1 $${\text{2NaOH }} + {\text{ CO}}_{{2}} \to {\text{ Na}}_{{2}} {\text{CO}}_{{3}} + {\text{ H}}_{{2}} {\text{O}}{.}$$

As the soil samples were carbonate free, there was no need to add barium chloride solution to precipitate carbonates. Columns with beakers were put into airtight bags (to ensure unchanged soil moisture and effective measurement of released CO_2_) and stored in an incubator at 20 °C. After one week of incubation, backtitration of the NaOH excess was performed with 0.5 M hydrochloric acid (HCl) by potentiometric titration (automatic titrator, Mettler Toledo, Inc. Columbus, Ohio). Backtitration was performed according to Eq. (2):

2 $${\text{NaOH }} + {\text{ HCl }} \to {\text{ NaCl }} + {\text{ H}}_{{2}} {\text{O}}{.}$$

The final result was presented as grams of CO_2_ released from a square meter of soil per day (g_CO2_ m^−2^ day^−1^).

### Laboratory analysis

After the soil CO_2_ respiration experiment, each column with soil was rinsed with 1 L of distilled water. The extracts obtained after filtration through filter paper were used to determine the concentration of DOC using a Shimadzu TOC-5000 analyzer (Kyoto, Japan). Afterwards, soil samples were air-dried at 60 °C for one week and then sieved through 2-mm mesh. The subsamples were ground by a ball mill (Fritsch, Idar-Oberstein, Germany) and were used to determine the concentration of C_t_ and nitrogen (N), using a LECO CNS TrueMac Analyzer (Leco, St. Joseph, Michigan, USA). Particle size distribution was measured using a laser diffraction analyzer Fritsch Analysette 22 (Idar-Oberstein, Germany).

The pyrophosphate extractable carbon (C_p_) concentration was determined after extraction of 3 mg of dried soil with 100 mL of 0.1 M sodium pyrophosphate decahydrate (Na_4_P_2_O_7_·10H_2_O) extractant^29^. Suspensions were shaken and stored for 16 h. Then, the extraction was performed, using a double filter (soft filter paper placed into hard filter paper). The C_p_ concentration was measured using a Shimadzu TOC-5000 analyzer. The soil pH was measured using a potentiometric method, with a combined electrode in a suspension of soil in distilled water, 1:5 mass-to-volume ratio, after 24 h of equilibration^29^. Total acidity (TA) was measured after 10 g of soil was extracted with 30 mL 1 M calcium acetate ((CH_3_COO)_2_Ca), shaken for 1 h and filtered. The samples on filters were rinsed with extractant solution up to volume 100 mL. 25 mL of the received extract was titrated by potentiometric titration (automatic titrator Mettler Toledo) to pH 8.2 with 0.1 M NaOH. Exchangeable sodium (Na^+^), magnesium (Mg^2+^), calcium (Ca^2+^) and potassium (K^+^) were extracted with 1 M ammonium acetate (CH_3_COONH_4_), pH = 7. Samples were mixed with an extractant (10 g of soil in 30 mL), shaken for 1 h and then equilibrated. Samples were filtered and filled with an extractant to a volume of 100 mL. The cation concentration was measured using an inductively coupled plasma optical emission spectrometry (ICP-OES Thermo iCAP 6500 Duo, Thermo Fisher Scientific, Cambridge, UK). Exchangeable aluminum (Al^3+^) concentration was estimated using ICP-OES after extraction of 3 g of soil sample with 30 mL of 0.5 M copper (II) chloride (CuCl_2_), which was shaken for 2 h.

### Statistical analysis

The data were divided into groups according to the sites, as well as the distance from the forest edge for statistical analysis. The mean and the standard error values for the investigated parameters were calculated for the research sites and the Kolmogorov–Smirnov (K–S) test was performed to assess significant differences between sites. The normality of the distribution of the variables was tested with the Shapiro–Wilk test. We tested the differences between the distances (2, 12, and 22 m from the forest edge) within the sites using ANOVA and Tukey’s HSD (honest significant difference) test. The Pearson correlation coefficients of the investigated properties were calculated, and principal component analysis (PCA) was conducted. Significance was defined at *p* < 0.05. All analyses were conducted using R statistical software^30^.

Given the high variation in the results from both sites, an analysis of the stoichiometry (ratio of the given variable to C_t_) was performed to standardize the parameters with the C_t_ concentration. First, the ratio between a given variable and C_t_ was calculated for each sample. The metal: C_t_ standardization expresses the saturation of SOM with the given metal. It allows to compare concentrations of SOM-associated metals and may compensate for the lack of data concerning the bulk density and metal pools^31–34^. The stoichiometry was calculated for the following characteristics: soil CO_2_ respiration, DOC, exchangeable base cations (Na^+^, Mg^2+^, Ca^2+^, K^+^) and exchangeable aluminum (Al^3+^).

## Results

Particle size distribution differed significantly between the investigated sites. Both sites were sandy; however, the proportions of the particle fractions differed (on average: pine site: sand 92%, silt 7%, clay 1%; fir site: sand 62%, silt 32%, clay 6%). The soil characteristics of the investigated sites are shown in Table 2.

**Table 2:** Chemical properties (mean values with standard error) of investigated soils.

Variable	Unit	Pine site	Fir site
C_t_	g kg^−1^	20.30 ± 0.60^a^	42.35 ± 3.72^b^
N		1.46 ± 0.03^a^	2.21 ± 0.10^b^
C_p_		3.14 ± 0.13^a^	3.72 ± 0.31^b^
pH	–	4.57 ± 0.08	4.59 ± 0.21
C_t_/N		13.87 ± 0.23^a^	18.92 ± 1.16^b^
DOC	mg L^−1^	127.64 ± 12.90	175.58 ± 23.29
Ca^2+^	cmol_(+)_ kg^−1^	1.41 ± 0.20	3.22 ± 0.97
K^+^		0.03 ± 0.003^a^	0.17 ± 0.02^b^
Mg^2+^		0.11 ± 0.01^a^	0.39 ± 0.06^b^
Na^+^		0.08 ± 0.01^a^	0.21 ± 0.02^b^
Al^3+^		2.24 ± 0.12^a^	3.38 ± 0.40^b^
TA		5.86 ± 0.35^a^	10.23 ± 1.31^b^
CEC		7.48 ± 0.32^a^	14.22 ± 1.20^b^

Different superscript letters indicate significant differences between sites (Kolmogorov–Smirnov test), *p* < 0.05.

*C*_*t*_ total carbon, *N* total nitrogen, *DOC* dissolved organic carbon, *C*_*p*_ pyrophosphate extractable carbon, *TA* total acidity, *CEC* cation exchange capacity.

The soil of both investigated sites was acidic, with average pH values of 4.5. However, except for the DOC and Ca^2+^ concentration, other properties differed significantly between sites (Table 2) (K–S test, *p* < 0.01). Overall, the measured chemical parameters were higher in soils under the fir stand.

Figure 2 shows the variability of the C fractions with the distance from the forest edge. The properties of soil under the fir stand changed clearly with proximity to the highway, whilst properties of soil under the pine stand varied less with distance.

Although the HSD test for C_t_ showed no significant differences between the distances in the fir stand, there was a noticeable positive trend with the distance from the forest edge (Fig. 2a). In the pine site, no trend was evident in C_t_ content; however, it differed significantly between the 12 and 22 m transects (mean ± standard error: 22.48 ± 0.33 g kg^−1^ and 18.29 ± 0.98 g kg^−1^, respectively). The C_p_ concentration (Fig. 2b) in the fir site showed a clear decrease near the forest edge (2 m), being significantly lower than at 12 m (2.55 ± 0.54 g kg^−1^ and 4.62 ± 0.32 g kg^−1^, respectively). In the soils of the pine site, the C_p_ concentration did not change much with distance. The content of DOC released from soil (Fig. 2c) in the fir site was significantly lower at 2 m compared with the other distances (2 m: 83.89 ± 18.04 mg L^−1^ < 12 m: 244.20 ± 39.79 mg L^−1^ ~ 22 m: 198.67 ± 19.40 mg L^−1^). In the pine site, no significant differences in DOC concentration between distances were found. Soil respiration showed no significant differences between distances in the fir stand (Fig. 2d), but in the pine stand, the respiration at 2 m was significantly higher than at 22 m (5.57 ± 0.47 and 3.83 ± 0.20 g_CO2_ m^−2^ day^−1^, respectively). In the fir stand, overall soil respiration was significantly higher (9.32 ± 0.28 g_CO2_ m^−2^ day^−1^) than in the soil of the pine stand (4.55 ± 0.28 g_CO2_ m^−2^ day^−1^).

**Figure 3:**
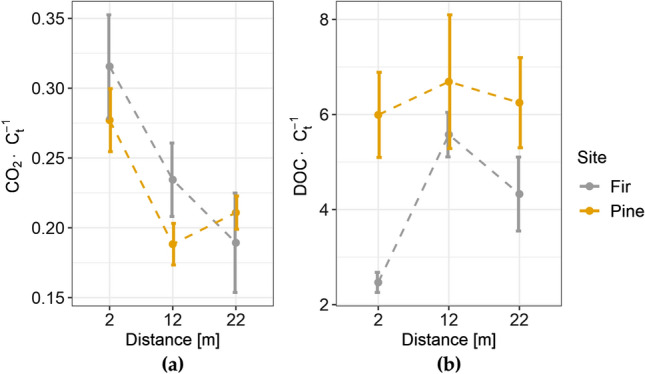
The soil respiration (**a**) and stoichiometric ratio of dissolved organic carbon (DOC) to total carbon (C_t_) (**b**), plotted against the distance from the forest edge. The circle represents the mean; the whiskers indicate the standard error.

The stoichiometric ratio of CO_2_ respiration to C_t_ in the fir stand was significantly higher in samples taken at 2 m (0.32 ± 0.04) compared with 22 m (0.19 ± 0.04) (Fig. 3a), whereas in the pine stand, the CO_2_:C_t_ in samples from 2 m was significantly higher than the other two distances (2 m: 0.28 ± 0.02 > 12 m: 0.19 ± 0.01 ~ 22 m: 0.21 ± 0.01). Overall, the CO_2_:C_t_ ratio was 0.25 ± 0.02 and 0.22 ± 0.01 for the fir and pine stand, respectively. A lower DOC:C_t_ (2.47 ± 0.21) at 2 m in fir stand is visible in Fig. 3b, and it differed significantly from the value at 12 m (5.58 ± 0.47). In the pine stand, the DOC:C_t_ ratio did not change significantly with distance.

**Figure 4:**
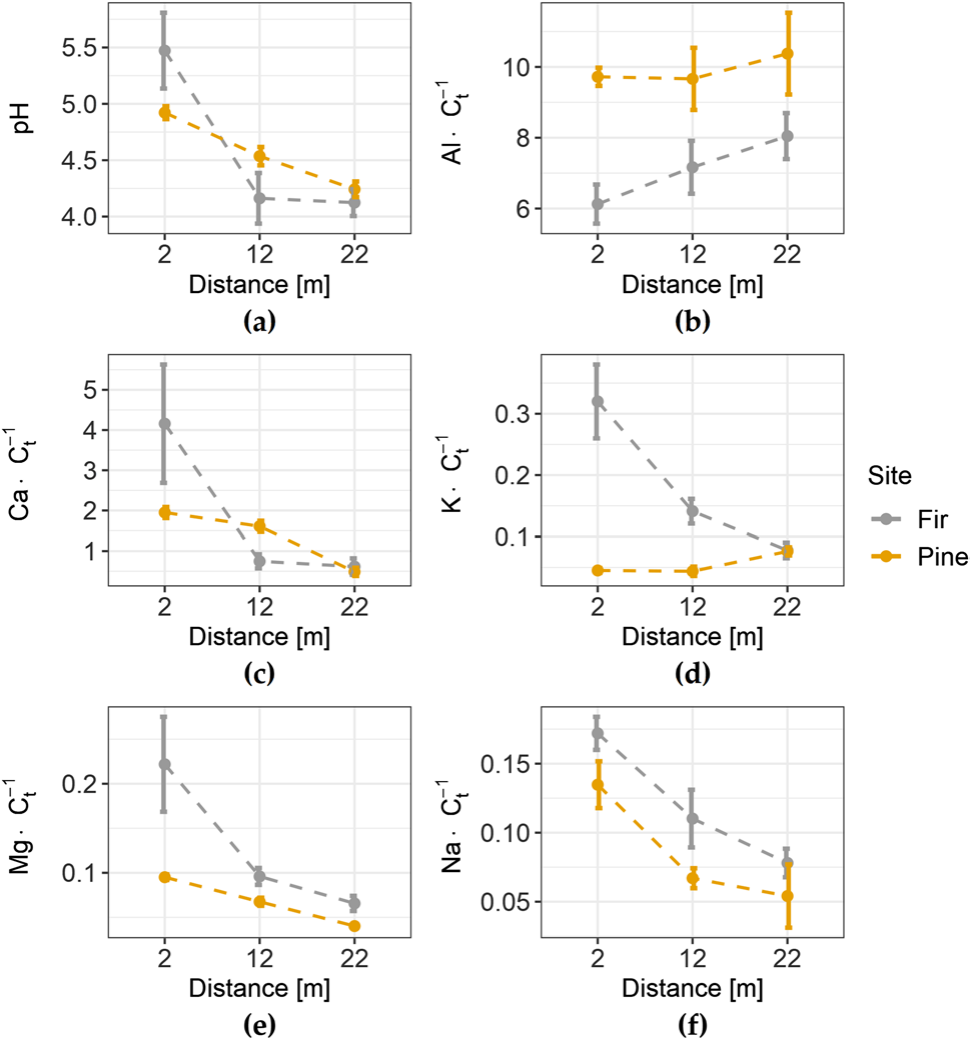
The soil pH (**a**) and stoichiometric ratios to total carbon (C_t_) of exchangeable aluminum (Al^3+^) (**b**), exchangeable base cations (Ca^2+^, K^+^, Mg^2+^, Na^+^) (**c**–**f**, respectively), plotted against the distance from the forest edge. The circle represents the mean; the whiskers indicate the standard error.

The soil pH, as well as the stoichiometric ratios of exchangeable base cations (Ca^2+^, K^+^, Mg^2+^, Na^+^) and exchangeable aluminum (Al^3+^) to C_t_, were calculated and plotted against the distance from the forest edge (Fig. 4). The soil pH was higher near the forest edge in both stands (Fig. 4a). According to the HSD test, pH at 2 m in the fir stand (5.47 ± 0.34) was significantly higher than at 12 m (4.16 ± 0.22) and 22 m (4.12 ± 0.12). In the pine stand, the pH decreased with increasing distance from the forest edge, and the average values differed significantly (2 m: 4.92 ± 0.06 > 12 m: 4.54 ± 0.08 > 22 m: 4.24 ± 0.07). The Al:C_t_ ratio in the pine stand showed no significant changes with the distance (Fig. 4b), whilst in the fir stand the Al:C_t_ ratio was significantly depleted at 2 m, compared with 12 m (6.13 ± 0.55 and 7.17 ± 0.75, respectively). The Ca:C_t_ ratio in the fir site (Fig. 4c) reached the highest value closest to the highway (2 m: 4.16 ± 1.47 > 12 m: 0.75 ± 0.17 ~ 22 m: 0.62 ± 0.20). In the pine site, the Ca:C_t_ ratios at 2 m (1.95 ± 0.14) and 12 m (1.61 ± 0.14) were significantly higher than at 22 m (0.49 ± 0.10). The K:C_t_ ratio (Fig. 4d) in the fir site at 2 m was considerably higher (2 m: 0.32 ± 0.06 > 12 m: 0.141 ± 0.02 ~ 22 m: 0.08 ± 0.01), whilst in the pine site the K:C_t_ ratios at 2 m and 12 m were significantly lower than at 22 m (2 m: 0.045 ± 0.004 ~ 12 m: 0.044 ± 0.008 < 22 m: 0.08 ± 0.01). The Mg:C_t_ ratio decreased with the distance from the forest edge in both sites (Fig. 4e). In the pine site, the Mg:C_t_ ratio of all distances differed from each other (2 m: 0.095 ± 0.003 < 12 m: 0.068 ± 0.005 < 22 m: 0.040 ± 0.002), whereas in the fir site the average values of the Mg:C_t_ ratio at 2 m (0.22 ± 0.05) differed from 12 m (0.10 ± 0.01) and 22 m (0.07 ± 0.001). The Na:C_t_ ratio was higher in close proximity to the forest edge in both sites (Fig. 4f). In both sites, the 2 m distance differed from 12 and 22 m (fir site: 2 m: 0.17 ± 0.01 > 12 m: 0.11 ± 0.02 ~ 22 m: 0.10 ± 0.01; pine site: 2 m: 0.14 ± 0.02 > 12 m: 0.07 ± 0.01 ~ 22 m: 0.05 ± 0.02).

To investigate the factors controlling soil CO_2_ respiration, we analyzed the relationships between soil respiration and other physiochemical soil properties using correlation analysis and principal component analysis (PCA). Pearson correlation coefficients were calculated for chemical properties standardized with C_t_. In the fir stand, significant (*p* < 0.05) positive correlation coefficients were observed between soil respiration and K:C_t_ (r = 0.71), Mg:C_t_ (r = 0.57), and pH (r = 0.76) and there was a negative correlation with the distance from the forest edge (r =  − 0.61). In the pine stand, CO_2_:C_t_ was significantly positively correlated with the Mg:C_t_ and Na:C_t_ ratios (r = 0.60 and r = 0.71, respectively), as well as with pH (r = 0.60) and negatively correlated with the distance from the forest edge (r =  − 0.53).

**Figure 5:**
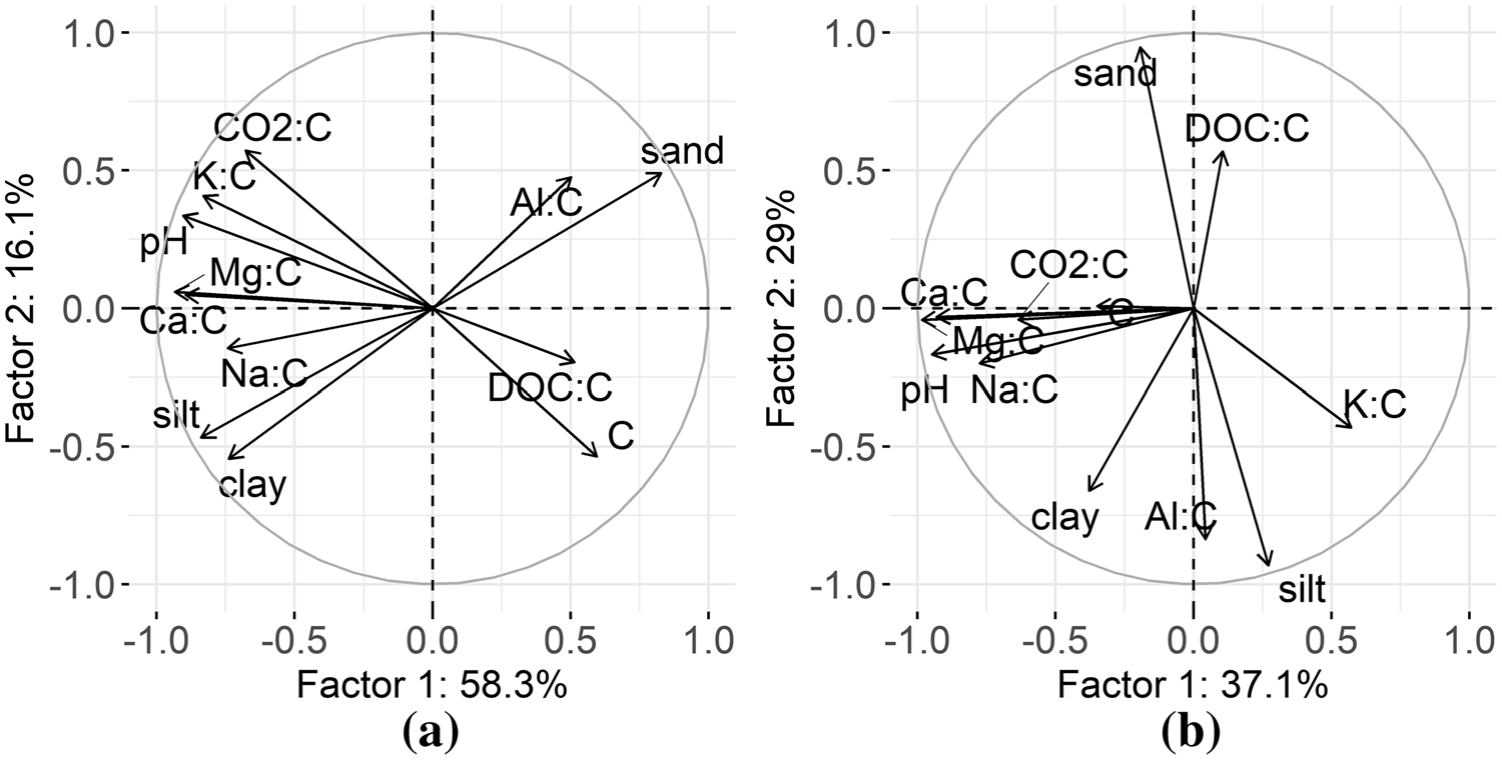
Two-dimensional projection of principal component analysis (PCA) for variables for soils in fir (**a**) and pine (**b**) stands.

The PCA was performed for the following variables: C_t_, DOC:C_t_, CO_2_:C_t_, Ca:C_t_, K:C_t_, Mg:C_t_, Na:C_t_, Al:C_t_, pH and the content (%) of sand, silt and clay to investigate the two-dimensional relationships between given variables, with an emphasis on soil CO_2_ respiration. The first two PCA variables for the fir stand explained 74.4% of the variance in total (Fig. 5a). Analysis showed that standardized soil respiration was positively related mainly with K:C_t_ and the pH. A negative relationship was observed for DOC:C_t_, and total C. Soil respiration in the fir stand was not related to particle size distribution and aluminum content. The first two PCA variables for the pine stand explained 66.1% of the variance in total (Fig. 5b). The relationships between soil respiration and other variables differed from the fir stand. The CO_2_:C_t_ was related positively with base cations, pH and C_t_. A weak negative relationship occurred for soil respiration and potassium content. No relationships were observed for the particle size distribution, DOC and Al content.

## Discussion

The chemical parameters presented in Table 2 indicate that the soils in the fir site had a slightly different texture than those in the pine stand, and also were more abundant in carbon and nitrogen. Organic matter is the main source of C_t_ and N in the top layers of the mineral horizons of carbonate-free soils. According to Gruba and Socha^14^, soil C stocks in pine stands are significantly lower than under fir stands in Polish soils. The main reason for the lower accumulation of C in the mineral horizons of pine stands is the limited capacity of these soils to sequester carbon, owing to their limited content of stabilizing factors such as clay and silt particles^8,14,35^. The C:N ratio from the fir site was higher than that of the pine site (18.9 and 13.9, respectively). However, in both cases, the ratio was less than 20, so it can be concluded that the litter decomposition conditions were relatively good in the soils of both stands.

Analysis of the spatial variation of total carbon concentration and its fractions (C_p_ and DOC) showed significant modifications near the forest edge (Fig. 2b,c), mainly in the fir site. Depletion of the C_t_ concentration in the fir stand nearer the highway corresponded with its fractions (C_p_, DOC). C_p_ represents the amount of humic and fulvic acids. The reduced concentration of these acids indicates much weaker stability of SOM. The stoichiometric ratio DOC:C_t_ also appeared to correspond with this observation (Fig. 3b). DOC is considered labile and has high mobility^36^. Hence, both low DOC concentration and low DOC:C_t_ ratio under conditions of reduced SOM stability may indicate an increased outflow of DOC in the vicinity of the highway. Higher soil CO_2_ respiration near the highway may also confirm the reduction in SOM stability in this zone. On the other hand, a rather small part of the SOM was mineralized to CO_2_ during the weekly measurement of soil respiration, so it may be not sufficient to conclude on the total SOM stability. The data on soil CO_2_ respiration after the one-week experiment can be interpreted as the biological activity of the soil, which was significantly increased in the vicinity of the highway. Information on the soil microbial biomass carbon and nitrogen would be useful in making conclusions, hence further research should focus on these properties. Previous studies suggest that soils close to roadways may be polluted^37–40^, thus CO_2_ respiration may be limited by heavy metals^41–43^. However, in our study sites significantly increased heavy metal concentration was not found, as shown in a separate paper^34^.

The effect of highway proximity on the ion exchange relationships of the soils studied appeared to be significant (Fig. 4). In both sites, the most pronounced influence was that of Na (from de-icing of the road), shown as Na:C_t_. The sodium content of soils from the study site was significantly increased in the zone up to 10 m from the forest boundary, as shown in a separate publication^34^. Also, the soil pH clearly increased in both sites in the vicinity of the highway. Saturation of SOM with Na is directly related to the winter de-icing process, during which mainly sodium chloride (NaCl) is applied. Another important effect of the influx of Na ions was their significant influence on the concentration of other ions and their stoichiometry, particularly Al. The significant increase in the Ca:C_t_ ratio at 2 m from the forest edge (Fig. 4c) in both sites may be due to the influx of Ca^2+^ ions from the road in part. Calcium chloride is an admixture used in de-icing salts, which is effective at temperatures below – 10 °C^20^. The increase in the Ca:C_t_ ratio, as well as the Mg:C_t_ ratio could also be coupled to the mobilizing effect of Na ions and their alkalizing properties. It is likely that Na^+^ competes with other cations for ion exchange sites, consequently leading to their temporal increase in the soil solution^39,44–47^. Increased levels of Ca and Mg correspond with previous studies showing that these elements occur in higher concentrations in close proximity to roadways^44,48^.

So far, the effect of ion exchange composition and its modification on the stabilization of soil organic matter is poorly established. Chemical stabilization of SOM with increasing saturation of H^+^ (protonation) at exchange sites on the SOM surface was previously suggested by Berggren et al.^49^. Protonation of SOM is strongly related to soil pH, thus, modification of the CEC composition and soil alkalization caused by Na^+^ ions will possibly influence SOM stability. Gruba and Socha^14^ found a relationship between the C stock and soil pH, based on generalized additive models (n = 468). In a large dataset of Polish samples from the 0–10 cm layer, the highest C stock was found at a soil pH of ~ 3.5–4.0 while the lowest stock was found at a pH of ~ 4.0–5.5. Previous studies^50,51^ have shown that at pH >  ~ 5 the proportion of exchangeable forms of Ca, Mg in the sorption complex gradually increases. This mechanism was observed on both study sites, but it was much more intense in the fir site, as shown by the stoichiometric ratios of these elements (Fig. 4).

Our data suggest that Na, Ca and Mg compete with Al, when introduced to soil, decreasing the Al:C_t_ ratio (Fig. 4b). This effect was pronounced in the soils of the fir site, where this ratio was significantly decreased near the forest edge, whereas in the pine stand no changes was found with respect to distance. This implies that in the soil of pine stands Na, Ca and Mg competed with H (increased pH) rather than with Al. This may be either because of smaller amounts of exchangeable Al (see Table 2) or the intrinsic cation-exchange properties of pine-derived SOM. Therefore, we suggest that the combined effect of Ca and Na ions, originating from winter de-icing, strongly influence the decreased proportion of H and Al in soil CEC. Aluminum, a key component of acidity, is considered by some authors to be responsible for SOM chemical stabilization^12,52^. It is also suggested that soil acidity can stabilize SOM through polyvalent cation-bridging between negatively charged SOM functional groups (phenolic, hydroxyl, carboxyl) and Al ions^53,54^. A relationship between Al and its immobilizing impact on SOM was described previously^13,55,56^. A linear positive correlation between soil acidity and C_t_ was shown by Mueller et al.^12^. In our study, a significant positive correlation between C_t_ and TA was found in the fir site (*p* < 0.0000, R^2^ = 0.83), whereas this relationship did not occur in the pine site.

Owing to significant differences in soil texture and carbon concentration, a direct comparison of these two sites is questionable. However, changes in the soil parameters with distance seemed to be related at least partly to the tree species. The sites differed in the changes (or not) with distance. The trends with the greatest difference were in the amounts of released DOC (Fig. 3b) and the Al:C_t_ ratio (Fig. 4b). The direct effect of stand species on the physical properties of soils is debated^12,57^. The most common view is that the influence of tree species is indirect and one of many factors. However, a contribution of tree species to the modification of CEC has been reported^12,56^. Intrinsic SOM properties (like hydrophobicity, the content of lignin-derived aromatic groups, other functional groups like alkyl or carbohydrate, the recalcitrance of bindings) derived from different tree species determine the chemical relationships between C_t_, CEC and soil pH^56^. The pine site showed less susceptibility to the modification of soil chemistry near the forest edge. We assume that the influence of different parent materials in both sites exerted more influence on the soil chemistry than the stand species composition. Pedogenic processes like the mineral weathering reactions can contribute to SOM stabilization through the release of hydrolyzing cations^12^. The relatively constant Al concentration with the distance from the forest edge in the pine site (Fig. 4b) and the subsequent greater SOM stability were most likely because of the parent material weathering process, during which polyvalent Al and Fe were released into the soil solution.

## Conclusions

The proximity of the S7 highway locally modified soil chemistry and significantly decreased the stability of SOM. The most visible effect of the highway vicinity was increased intensity of soil CO_2_ respiration, mainly expressed as the CO_2_:C_t_ ratio, in the soils of both research sites. The increased CO_2_ respiration may be interpreted as higher soil biological activity rather than decreased total SOM stability. On the other hand, the data about the soil CO_2_ respiration with both the C_p_, representing humic and fulvic acids, and the DOC decreased in closer proximity to the highway, imply lower stability of SOM. Further research on this issue should be oriented towards soil microbial biomass carbon and nitrogen. The Ca^2+^ and Na^+^ ions, originating from winter road de-icing and migrating from the road surface, modified the CEC composition of the adjacent forest soils, mainly by increasing the soil pH. We suggest that the combined effect of Ca^2+^ and Na^+^ ions caused the depletion of Al and H in the soil close to the forest edge and, therefore, resulted in lower stability of SOM expressed as the release of CO_2_, C_p_ and DOC. Therefore, we conclude that the changes of SOM stability with distance were the effect of modification of ion-exchange relationships (particularly base cations versus Al^3+^ with H^+^) rather than forest stand species or intrinsic properties of SOM (like functional groups, the recalcitrance of bindings etc.), derived from pine or fir stands. Our work supports earlier studies, confirming the significant impact of Al and H on SOM stability.

## Data Availability

The datasets generated during the current study are available from the corresponding author on reasonable request.
